# Experimentally Observed Nucleation and Growth Behavior of Mg/MgH_2_ during De/Hydrogenation of MgH_2_/Mg: A Review

**DOI:** 10.3390/ma15228004

**Published:** 2022-11-12

**Authors:** Jinzhe Lyu, Viktor Kudiiarov, Andrey Lider

**Affiliations:** 1 Division for Experimental Physics, School of Nuclear Science & Engineering, National Research Tomsk Polytechnic University, Lenin Ave. 43, 634050 Tomsk, Russia; 2 School of Electrical and Mechanical Engineering, Pingdingshan University, Pingdingshan 467000, China

**Keywords:** nucleation and growth, hydrogen, magnesium, magnesium hydride

## Abstract

With the increasing energy crisis and environmental problems, there is an urgent need to seek an efficient renewable energy source, and hydrogen energy is considered one of the most promising energy carriers. Magnesium is considered a promising hydrogen storage material due to its high hydrogen storage density, abundant resources, and low cost. However, sluggish kinetic performance is one of the bottlenecks hindering its practical application. The kinetic process of hydrogenation/dehydrogenation can be influenced by both external and internal factors, including temperature, pressure, elementary composition, particle size, particle surface states, irregularities in particle structure, and hydrogen diffusion coefficient. The kinetic performance of the MgH_2_/Mg system can be effectively improved by more active sites and nucleation centers for hydrogen absorption and desorption. Herein, we briefly review and discuss the experimentally observed nucleation and growth behavior of Mg/MgH_2_ during de/hydrogenation of MgH_2_/Mg. In particular, the nucleation and growth behavior of MgH_2_ during the hydrogenation of Mg is discussed from the aspect of temperature and hydrogen pressure.

## 1. Introduction

**Figure 1:**
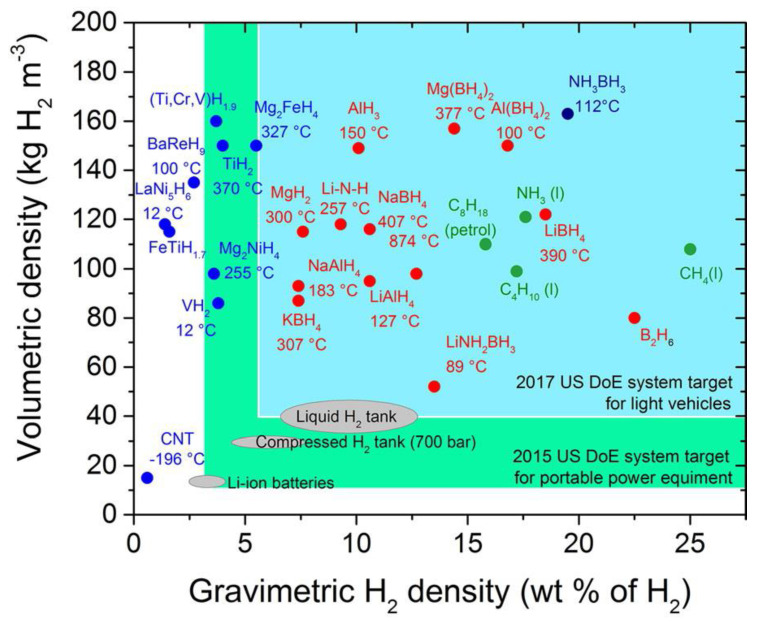
Potential high–capacity hydrides [43].

Compared with the utilization of fossil energy, hydrogen does not emit CO_2_ gas that causes global warming after combustion or the reaction of generating electricity in fuel cells. The pollution-free nature and huge reserves on earth make hydrogen energy known as one of the best alternatives to fossil fuels [1,2,3,4,5,6,7,8,9]. To achieve the effective development and application of hydrogen energy, especially in the growing market for mobile devices and unmanned aerial vehicles, both of which require small-sized energy sources based on fuel cells, the three problems of clean hydrogen production, compact storage, and efficient transportation need to be solved [10,11,12,13,14]. As per the guidelines of the United States Department of Energy (DOE), by 2025, gravimetric and volumetric storage capacities are required to meet the target of 5.5 wt% and 40 g/L, at temperatures in the range of −40–60 °C and pressures up to 10 MPa [5]. Among many lightweight and high-capacity hydrogen storage materials (Figure 1) [15,16,17,18,19,20,21,22], magnesium is favored because of its theoretical hydrogen storage capacity of up to 7.6 wt% (110 kg/m^3^), abundant resources, and low cost [13,23,24,25,26,27]. However, its application is limited by its high dehydrogenation temperature (>300 °C) [28,29,30]. Moreover, sluggish hydrogen absorption and desorption kinetics are observed due to the fact that the hydrogen absorption and desorption reactions of the MgH_2_/Mg system involve different gas-solid reaction energy barriers up to 218 kJ/mol, including hydrogen dissociation, hydrogen diffusion, and nucleation and growth processes [29,31,32,33]. The hydrogen absorption process in metallic Mg has been divided into the following steps [31,34,35,36,37,38]: (1) physisorption of hydrogen molecules on the surface of metallic Mg; (2) dissociation of hydrogen molecules and chemisorption. In this step, H_2_ dissociation can be influenced by surface properties, including morphology, surface structures, and the purity of the Mg; (3) surface penetration and bulk diffusion of hydrogen atoms. In this stage, the variation in the microstructures of Mg, such as the grain size and grain boundaries, may cause a significant difference in the diffusion process of H atoms inside the metal; (4) formation of a solid solution (*α*-phase) as a result of the diffusion of hydrogen atoms into the interstitial sites of the Mg lattice. Dislocations, vacancies, and other microstructure defects exert a significant influence at this stage. For example, each vacancy can capture up to six H atoms with large binding energy; (5) formation of a saturated solid solution due to the continuous diffusion of hydrogen atoms into the interstitial sites of the Mg lattice, followed by the formation of MgH_2_ phase/nuclei (*β*-phase) due to the reaction between the excess hydrogen atoms and the solid solution; (6) diffusion of hydrogen atoms through the MgH_2_ layer; and (7) hydride growth at the Mg–MgH_2_ interface. The hydrogen desorption process is the reverse of hydrogen absorption. It should be noted that the transformation from the *α*-phase to the *β*-MgH_2_ is not one step. Hydrogen absorption leads to volume expansion of the hcp Mg lattice, which exists in a very narrow hydrogen concentration range [39,40]. With further increases in hydrogen concentration, a phased transformation from the hcp structure of MgH*x* to the fcc structure of MgH*x* occurs with the eventual formation of bct MgH_2_ [40,41]. It can be believed that these transformations on hydrogenation can be affected by both external and internal factors and thereby influence the nucleation and growth of the *β*-MgH_2_. Unfortunately, experimentally only the initial (*α*-phase) and final states (*β*-MgH_2_) can be caught, making the internal fcc structure of MgH*x* impossible to survey [42].

Many published articles [44,45,46,47,48,49,50] focused on the introduction of kinetic models used to fit experimental hydrogen absorption and desorption data of Mg rather than the scope of application of kinetic models according to kinetic measurement methods. However, the kinetic mechanism of the hydrogen absorption and desorption reactions in Mg is not always the same. This is related to the preparation methods and kinetic test conditions of hydrogen absorption and desorption, which influence the kinetic process of hydrogenation/dehydrogenation by different external and internal factors, including temperature, pressure, elementary composition, particle size, particle surface states, irregularities in particle structure, and hydrogen diffusion coefficient [48,51]. Different from the external factors, the influence of which can be expressed as activation energy and pressure terms in the theoretical kinetic models, the influence of the internal factors on hydrogenation and dehydrogenation is always represented by the rate constant in many kinetic models due to the difficulty of explicitly expressing these factors in the kinetic models [51]. However, the volume expansion and contraction of particles during the hydrogen absorption and desorption processes could influence the prediction accuracy of the kinetic models for metal hydrides with large volume changes [51]. Thus, there is a great deal of debate surrounding the kinetic mechanism of the hydrogen absorption and desorption reactions in Mg. Although the effect of driving forces on the rate-limiting steps is still not fully illustrated [52], the rate-limiting steps are generally considered to be nucleation and growth processes controlled by H-diffusion in the MgH_2_ phase for absorption and interface reactions for desorption [51,53,54]. Thus, in this review, we present a qualitative analysis of the influence of both external and internal factors on the experimentally observed nucleation and growth behavior of Mg/MgH_2_ during de/hydrogenation of MgH_2_/Mg. We believe this review is helpful for researchers who need to quickly choose accurate parameters in kinetic models, especially in the nucleation and growth model, according to kinetic measurement methods for Mg/MgH_2_.

## 2. The Nucleation and Growth Behavior of Mg Crystallites during Hydrogen Desorption of MgH_2_

**Figure 2:**
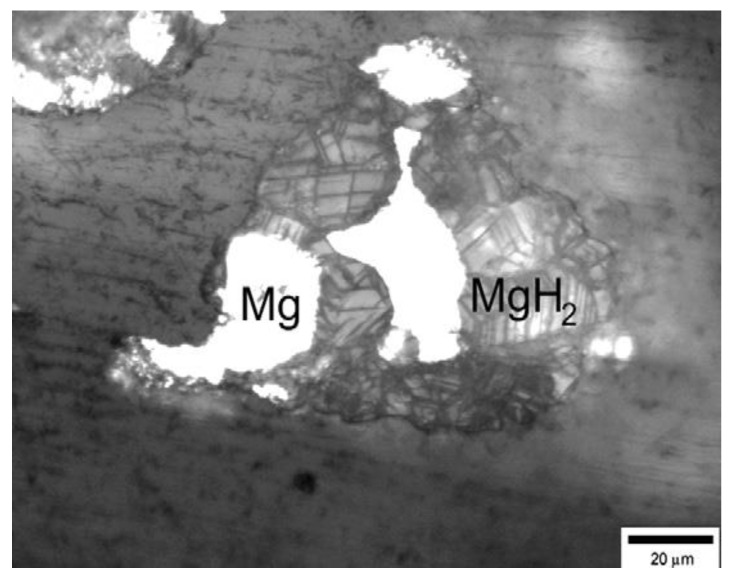
Optical microscopy photograph of the etched metallographic section of a partially decomposed magnesium hydride (approximate bulk composition of the sample MgH_1.3_) [57].

The hydriding/dehydriding mechanisms of MgH_2_ have long been the subject of debate [55]. Various kinetic models for the hydriding/dehydriding of MgH_2_ have been developed, such as the “shrinking core” mechanism [45], “nucleation and growth” mechanism [46], “multiple step” mechanism [45,46], “migration and coalescence” (Greenwood and Speight) model [55], and the Ostwald ripening model [55]. For a more detailed description of the kinetic mechanism of the hydrogen absorption and desorption reactions in Mg, one can refer to the review [56]. It was interesting to note that the “shrinking core” and “nucleation and growth” models describe two quite different MgH_2_ desorption behaviors. In the former, the hydrogen desorption process begins with the Mg skin formation surrounding MgH_2_, followed by the shrinkage of the MgH_2_ core region. At the same time, the latter holds that the nucleation of Mg, randomly proceeding within MgH_2_, starts the hydrogen release, and the growth of Mg along Mg nuclei continues the transition [46]. Stepura et al. [45], using the model based on the shrinking core approach, successfully predicted the TGA (thermogravimetric analysis) and DTA (differential thermal analysis) test curves of magnesium hydride decomposition with a mean particle size of 0.5 μm. TDS (thermal desorption spectroscopy) results in the work of Evard et al. [57] supported the fact that the nucleation of Mg does not occur until higher operating temperatures are used. Hydrogen release from MgH_2_ occurs only when the Mg nucleus appears on the surface of the MgH_2_ powder particles. This is consistent with the findings that the desorption of hydrogen from both milled and unmilled pure MgH_2_ is controlled by a slow nucleation and growth process below 350 °C, even though the driving force for desorption is great at these temperatures [58]. Furthermore, Evard et al. [57], based on the optical microscopy studies (Figure 2) of incompletely decomposed MgH_2_, judged the inappropriateness of the “shrinking core” model. As can be seen from Figure 2, during desorption, the Mg islets (light regions) appeared and grew afterward from the surface into the bulk MgH_2_ (dark regions). Meanwhile, on the basis of the presented data in Figure 2, Evard et al. [57] conclude that for the stoichiometric MgH_2_ particles, hydrogen desorption consists of two individual stages: (1) Nucleation of the Mg “windows” on the particle surface; (2) Hydrogen release through the Mg “windows” acting as hydrogen channels.

**Figure 3:**
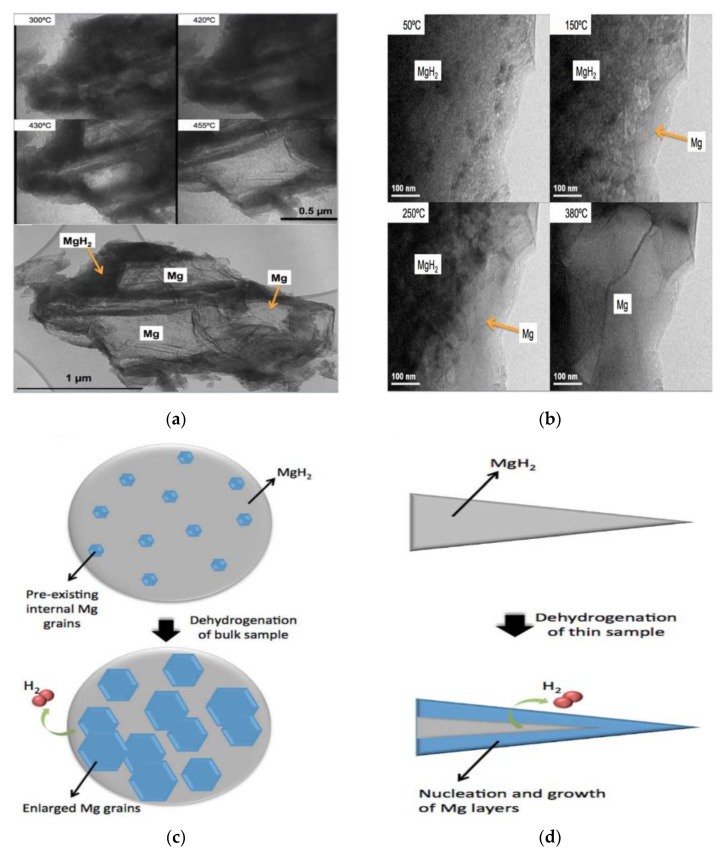
(**a**) Selected still frame TEM images from in situ video of high voltage (1000 kV) TEM of a ~2 μm bulk MgH_2_ particle taken at 300 °C, 420 °C, 430 °C, and 455 °C, and a low magnification bright field image of the sample (a single bulk powder particle) at 455 °C (bottom) [55]; (**b**) Selected still frame TEM images from in situ videos of conventional (200 kV) TEM through a thinned region (a few tens of nm) of an MgH_2_ particle taken at 50 °C, 150 °C, 250 °C, and 380 °C [55]; (**c**) Schematic multiple “nucleation and growth” hydrogen release mechanisms for bulk MgH_2_ grains [55]; (**d**) Schematic “shrinking core” hydrogen release mechanisms for thin MgH_2_ TEM samples [55].

Gabis et al. [59] believed that for the dehydrogenation of non-metallic (ion-covalent) hydrides, the morphology of “nucleation and growth” is typical due to the fact that only a few nuclei appear relatively slowly and rarely form a skin as a result of the slow (compared to metals) hydrogen desorption, while for metallic ones, the “shrinking core” morphology is more common due to the fact that so many nuclei of the new phase appear and later form a solid skin of the new phase as a result of the fast desorption from the entire surface of the metal parent phase. This can lead to two suggestions: (1) The dehydrogenation of MgH_2_ should be controlled by the “nucleation and growth” mechanism because MgH_2_ is a semiconductor with a relatively large energy gap of 4.16 eV [60,61,62]; (2) The nucleation rate of Mg from MgH_2_ is the major cause that poses the argument between the “nucleation and growth” mechanism and the “shrinking core” mechanism, i.e., the difference in the nucleation rate in various MgH_2_ dehydrogenation experiments led to the fact that some experimental results were successfully explained by the “nucleation and growth” model while others by the “shrinking core” model. The evidence for the rationality of the last suggestion may also be served by the finding of Nogita et al. [55]. They performed an in situ ultra-high voltage transmission electron microscopy (TEM) on the Mg-Ni alloys to directly verify the hydrogen desorption mechanisms for MgH_2_. It was found by the authors [55] that the hydrogen desorption of bulk (2 μm) MgH_2_ particles proceeds as a result of the growth of multiple pre-existing Mg crystallites (nuclei) present due to the difficulty of the full transformation of all Mg during a hydrogenation cycle within the MgH_2_ matrix without the formation of new nuclei of Mg phase on the surface (Figure 3a,c). This agrees with the mechanism proposed by Evard et al. [57] for the desorption process of the partially hydrogenated magnesium. In contrast, in thin samples analogous to nano-powders (Figure 3b,d), hydrogen desorption occurs by a “shrinking core” mechanism.

**Figure 4:**
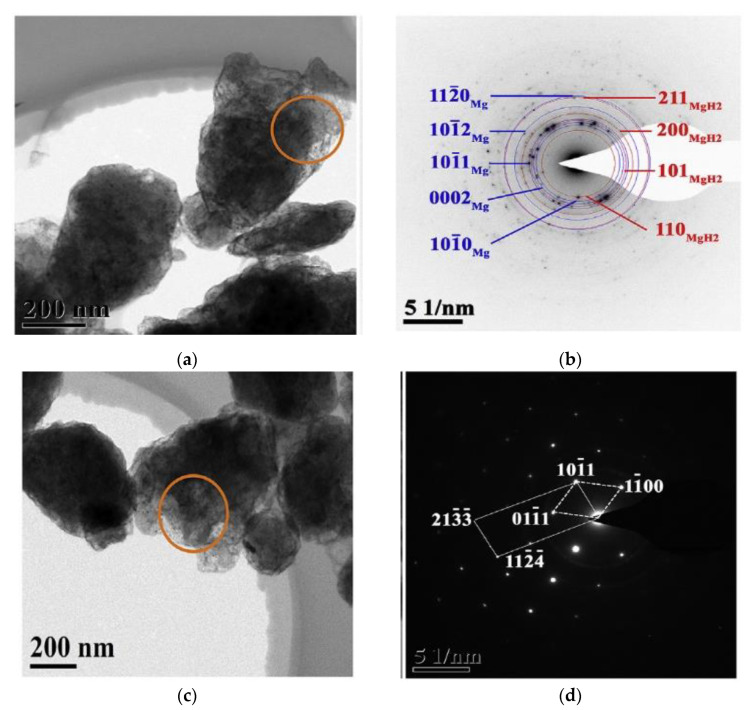

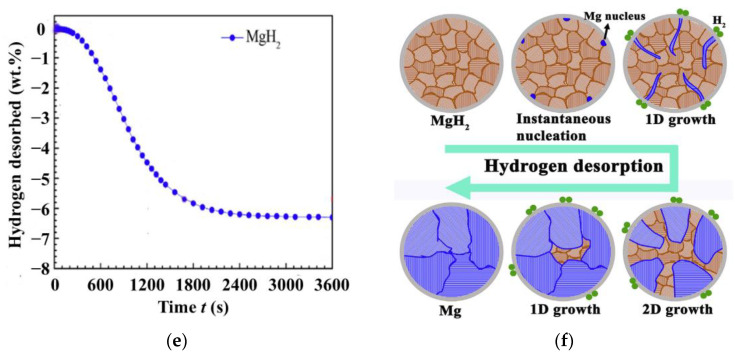
(**a**) TEM images for the partially dehydrogenated MgH_2_ samples [46]; (**b**) corresponding SAED patterns of (**a**) [46]; (**c**) TEM images for the entirely dehydrogenated MgH_2_ samples [46]; (**d**) corresponding SAED patterns of (**c**) [46]; (**e**) Isothermal dehydrogenation curves of MgH_2_ samples at 623 K [46]; (**f**) Schematic illustration of the growth mechanism of Mg crystallites during hydrogen desorption of MgH_2_ [46].

Thus, despite the argument about the dehydrogenation models of MgH_2_, the dehydrogenation of MgH_2_ is generally considered to be the “nucleation and growth” process, which can be transformed into its extreme form—”shrinking core” mechanism, when the nucleation rate is quite high. The Johnson-Mehl-Avrami-Kolmogorov (JMAK) equation, which is based on the “nucleation and growth” mechanism, allows a good description of the dehydrogenation kinetics of MgH_2_. Even if the JMAK model is based on the assumption of homogeneous nucleation through a bulk sample, clearly heterogeneous nucleation of Mg at the free surface of MgH_2_ can be well fitted by the JMAK equation [63]. Recently, based on the results of TEM images (Figure 4a,c) and corresponding selected area electron diffraction (SAED) patterns (Figure 4b,d) for the partially (Figure 4a,b) and entirely (Figure 4c,d) dehydrogenated MgH_2_ samples, which support the “nucleation and growth” mechanism for the desorption of nanocrystalline MgH_2_, Zhou et al. [46], using the JMAK equation, fitted experimental data for pure MgH_2_ (Figure 4e) in different stages of isothermal dehydrogenation at 623 K. Based on the modeling results, the authors [46] proposed three stages for the “nucleation and growth” mechanism of Mg crystallites during the dehydrogenation of MgH_2_ (Figure 4f): (1) Instantaneous nucleation of Mg crystallites at the free surfaces of particles followed by the one-dimensional (1D) interface-controlled growth of Mg crystallites; (2) Two-dimensional (2D) growth of Mg crystallites. In this stage, the hydrogen desorption proceeds relatively quickly due to the interface-controlled thickening of linear Mg crystallites, which do not stop until the interconnection of neighboring crystallites with each other; (3) The longitudinal direction (1D) growth of Mg crystallites towards the end of the transition, which leads to a slower and slower hydrogen desorption rate.

**Figure 5:**
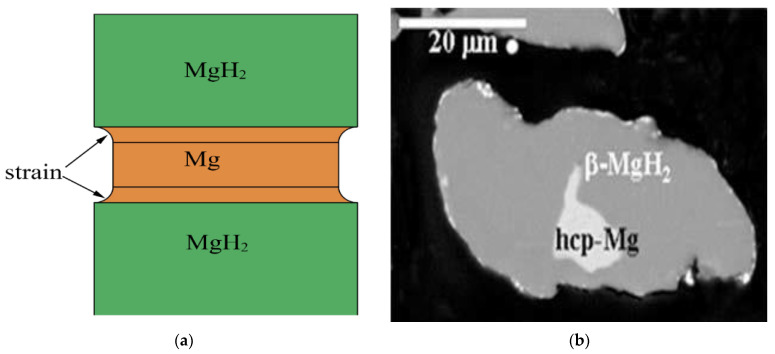
(**a**) Schematic nucleation of Mg within the volume of pure MgH_2_; (**b**) SEM (scanning electron microscopy)/BSE (backscattered electron microscopy) micrographs of the saturated powder revealing the impingement of the *β*-MgH_2_ colonies [66].

According to the above discussion, the “nucleation and growth” behavior during the dehydrogenation of pure MgH_2_ can be summarized as follows: (1) At low temperatures, the rate of dehydrogenation of MgH_2_ is slow due to the low nucleation rate. Thus, the rate-limiting step at this stage is nucleation; (2) As the temperature rises to a certain value, metallic Mg nucleates instantaneously at the free surfaces of particles; (3) Decomposition of MgH_2_ and growth of Mg at the Mg–MgH_2_ boundary (the movement of the inter-phase boundary), which is derived from the free energy difference between atoms in adjacent grains [55]; (4) The hydrogen atoms generated at the boundary diffuse through Mg to the surface of Mg. For ion-covalent hydrides, both ways (through metal or semiconductor) are possible. However, diffusivity in the magnesium hydride is approximately three orders of magnitude less than that in metallic magnesium. At the same time, its activation energy is significantly higher due to the fact that the rate of diffusion increases as the concentration of free charge carriers does, so the main diffusion flux of hydrogen from the phase boundary to the outer surface is through the metallic phase [59,64]. If the mean diffusion path *L* = 10 μm, then the typical diffusion time *τ* = *L*^2^/*D* for magnesium at 400 °C is *τ* = 0.03 s [59]. This means that the gradient of concentration of hydrogen dissolved in metallic magnesium is very low, while its diffusion is fast, it can hardly significantly influence the total rate of hydrogen evolution [59]; (5) Desorption of hydrogen atoms, which is the rate-limiting step at high temperatures [57]. It was suggested that in the stage of fast hydrogen desorption, the surface recombination required for the formation of the H_2_ molecule is not fast enough and represents the rate-limiting step, while only later when the reaction is slower, does the reaction rate at the Mg–MgH_2_ interface become a rate-limiting step [63]. It should also be noted that the spatial distribution of any pre-existing Mg nuclei, and in particular, the distance of these nuclei from the free surface, will play an important role in determining the “nucleation and growth” behavior and, thereby, the desorption rate of the bulk samples. It has been confirmed that for pure MgH_2_ without pre-existing Mg grains, surface nucleation of Mg (e.g., Figure 2, Figure 3b,d and Figure 4f) would be easier than nucleation of Mg within the volume due to the volume change [55,65,66], which leads to more strain for nucleation of Mg within the volume than that for surface nucleation of Mg, as can be seen in Figure 5a.This implies that the presence of the Mg phase on the surface of MgH_2_ matrix can facilitate the elimination of the nucleation barrier for the formation of the Mg phase from the MgH_2_ matrix [57,67]. However, there is a great deal of debate surrounding the situation in the presence of pre-existing Mg grains within the volume (Figure 5b). Some results showed more favorable growth from within than nucleation and growth from the surface in the presence of pre-existing Mg grains within the volume (e.g., Figure 3a,c) [55,65]. In contrast, there is another opinion suggesting that when the surface of the powders is completely covered with the MgH_2_ phase, the observable dehydrogenation process most probably begins with the nucleation of the hcp-Mg on the surface of the hydrogenated powders as a result of the extremely slow rate of growth of the hcp-Mg phase left in the core of the particles owing to the low diffusivity of hydrogen through the MgH_2_ phase [66]. We believe that the low diffusivity of hydrogen through the MgH_2_ phase can also serve as one of the reasons for easier surface nucleation than nucleation within the volume of pure MgH_2_ without pre-existing Mg grains.

## 3. The Nucleation and Growth Mechanism of MgH_2_ Crystallites during Hydrogenation of Mg

**Figure 6:**
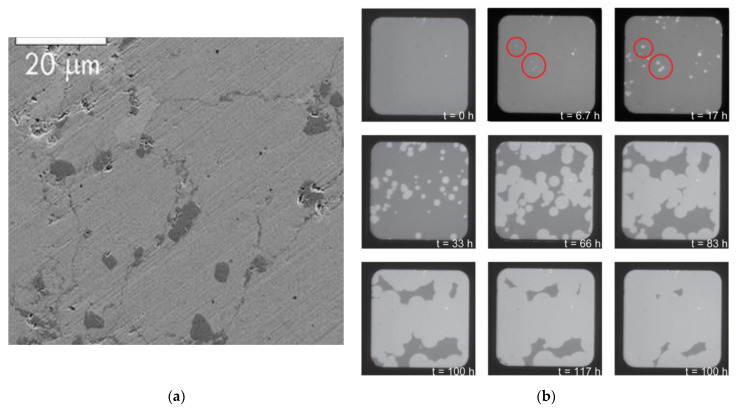
(**a**) SEM images at 1 kV at different magnifications of the MgH_2_, where nucleation of MgH_2_ phase (the dark particles) occurs at grain boundaries [63]; (**b**) Visual images of the Ti–Mg–Ti–Pd multilayer sample hydrogenation at several times during a full hydrogenation cycle under hydrogen pressure of 70 Pa and temperature of 90 °C. The MgH_2_ nuclei are indicated by the red circles [79].

**Figure 7:**
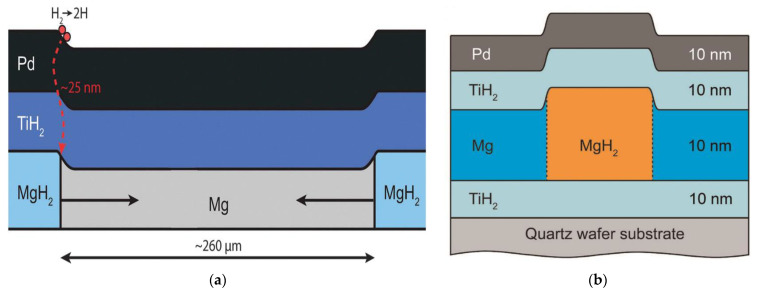
Schematic representation of the deformation taking place during hydrogenation with a nucleated MgH_2_ domain (**a**) at the edges [79] and (**b**) in the middle [78], due to the 30% volume expansion of MgH_2_.

**Figure 8:**
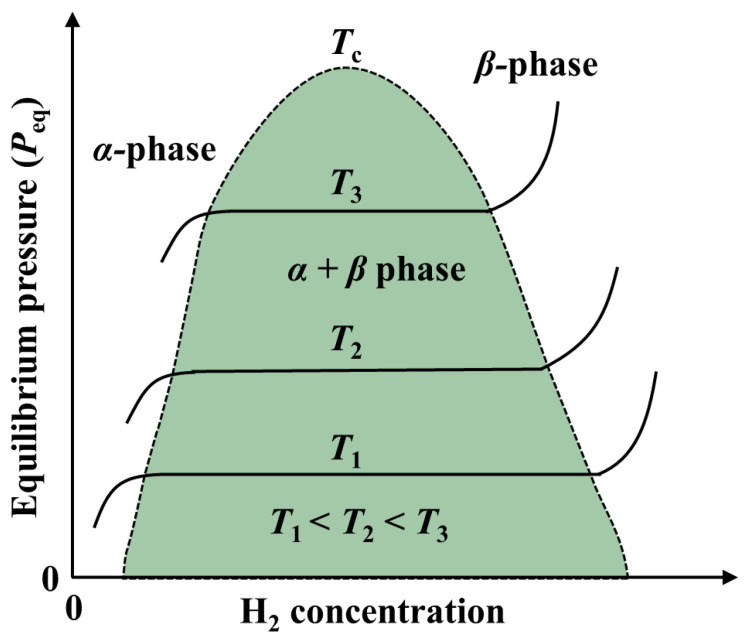
Pressure composition isotherm (PCI) plot of hydrogen-metal systems [85].

The nucleation behavior and its influences on Mg hydrogenation and MgH_2_ dehydrogenation are asymmetric: (1) In the process of hydrogenation of pure Mg, both the surface (Figure 6a) and the volume (Figure 6b) can serve as the nucleation sites of MgH_2_ [63,68] due to the fact that the hydrogen diffusion coefficient of Mg is higher than that of MgH_2_ [4,55,63] and MgH_2_ nucleation in Mg can be assisted by crystal defects present throughout the matrix (Figure 6a) [63,69,70,71], which are perceived as essential to dispel the accumulated elastic strain during the phase transformation [72,73,74] since around 20~31% expansion (Figure 7) of the volume of the initial Mg metal occurs to form the rutile-type tetragonal phase of MgH_2_ [53,75,76,77,78,79]. Furthermore, it was also suggested that the volume expansion induced defects in the hydrogenation process [80] lead to fast and easy nucleation and growth of the *β*-phase [81]; (2) The hydrogen storage capacity and absorption rate of Mg significantly depend on the driving force for MgH_2_ nucleation, which is proportional to the deviation from the equilibrium condition [82]. At constant temperature, the hydrogen pressure-induced driving force is related to the equilibrium plateau pressure of the pressure-composition isothermal (PCI) curve [52]. It has been experimentally demonstrated that the MgH_2_ nucleation rate during the hydrogenation of Mg is low when the hydrogen pressure nears the equilibrium plateau pressure [82]. More specifically, as can be seen from Figure 8, the nucleation rate of *β*-MgH_2_ during the hydrogenation is reduced with a decrease in pressure [71,83,84] and increased with a decrease in hydrogenation temperatures [71,82].

**Figure 9:**
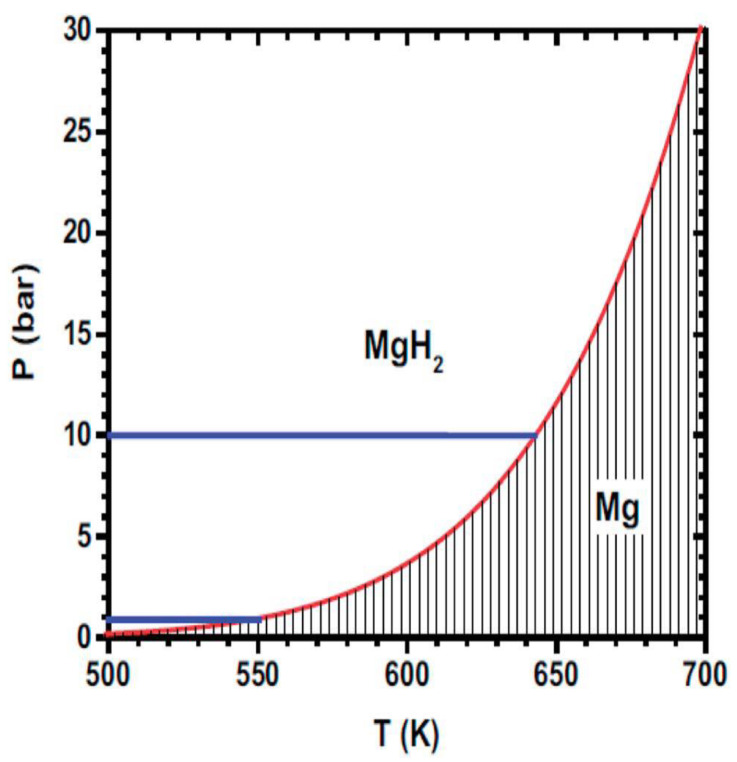
The variation of plateau pressure as a function of temperature for magnesium hydride [24].

**Figure 10:**
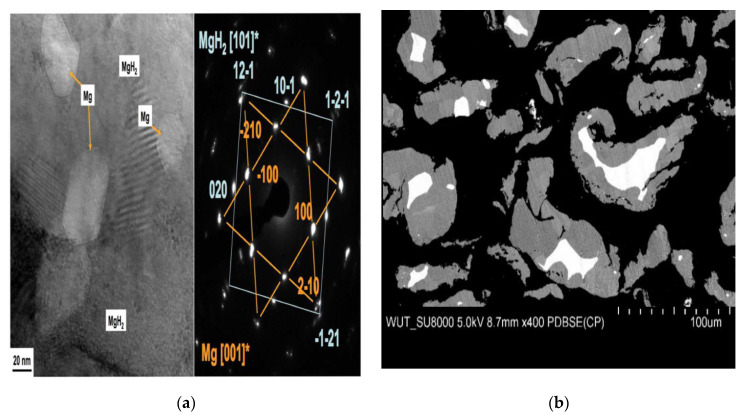
(**a**) A TEM image and selected area electron diffraction patterns from Mg and MgH_2_ phases in a nominally fully hydrogenated bulk MgH_2_ (hydrogen absorption at 350℃ and 2 MPa for 20 h) [55]; (**b**) Cross-section of commercially available magnesium hydride particles with visible white magnesium cores [88].

Thermodynamically, the reaction of metallic Mg with hydrogen should proceed at notably low pressure (<1 bar) and low temperatures (below 50 °C) [24,86,87], which can be supported by Figure 9. In practice, however, the reaction between metallic Mg and hydrogen is not observed at low temperatures, even at 100-bar hydrogen pressure [86]. Due to slow kinetics, the conversion from magnesium to magnesium hydride is very difficult below 350 °C, even when the magnesium is prepared into a very fine powder [88,89]. Thus, conventionally, the hydrogenation of Mg without additives requires temperatures and pressures as high as 350 °C and 70 bar H_2_ [90]. This phenomenon arises from the fact that an oxide passivation layer can be easily formed on Mg even when Mg is stored in a globe box [38,91,92,93,94]. This induces an extremely high activation energy barrier (2.34–2.94 eV or 226–284 kJ mol^−1^) to the dissociative adsorption of hydrogen [75] and may hinder the penetration of H atoms, thereby decreasing the hydrogen nucleation and growth of MgH_2_ in Mg [35,95,96,97] due to the weak nature of the interactions between H_2_ and magnesium oxide clusters (the hydrogen physisorption induced by the electrostatic field, which is produced by the polarity of the Mg–O bond due to the charge transfer from the magnesium atoms to the oxygen ones) [98]. Hence, Mg requires initial activation to absorb hydrogen in order to induce the passivation film cracks so that bare Mg surfaces are accessible to hydrogen [99]. However, even after activation, the sorption kinetics can still be rather sluggish [88]. This is due to the so-called “blocking effect” of the MgH_2_ layer [41,100]. The diffusion coefficient or diffusivity (*D*) of hydrogen in MgH_2_ is low, down to 10^−18^ m^2^ s^−1^ at 300 °C [53,101,102,103], which is at least three orders of magnitude less than that in Mg [4,55,64]. Thus, as the hydrogenation reaction progresses, a hydride layer that grows on the Mg surface limits the ability of hydrogen atoms to diffuse into the volume [50,104,105,106]. The low diffusion coefficient of H_2_ in MgH_2_, on the one hand, is another aspect of the sluggish kinetics [4]. On the other hand, it can serve as one of the factors leading to the issue of incomplete hydrogenation of bulk Mg due to the fact that the growth of hydride colonies/grains leads to a decrease in the total effective cross-section area for hydrogen diffusion into the magnesium phase. After the impingement of the hydride colonies/grains, the growth of hydride is limited by the hydrogen diffusion through the hydride and maximum capacity is reached [82]. Even now, it is difficult to find commercial magnesium hydride with a purity of more than 90% (despite the official specifications given by manufacturers) [88]. The issue of incomplete hydrogenation of bulk Mg is also believed to be related to the strain energy inhibiting the growth of MgH_2_ as MgH_2_–Mg interfaces grow into the last small MgH_2_-surrounded Mg islands (Figure 10a) [55].

Thus, the composition of magnesium hydride usually involves magnesium hydride, magnesium metal, which is present in the core of the particles [52] (Figure 10b), and magnesium hydroxide contamination, which is usually present in the form of an amorphous layer on the surface of the particles [88]. However, it should be noted from Figure 10b that some Mg particles with small sizes are completely hydrogenated.

**Figure 11:**
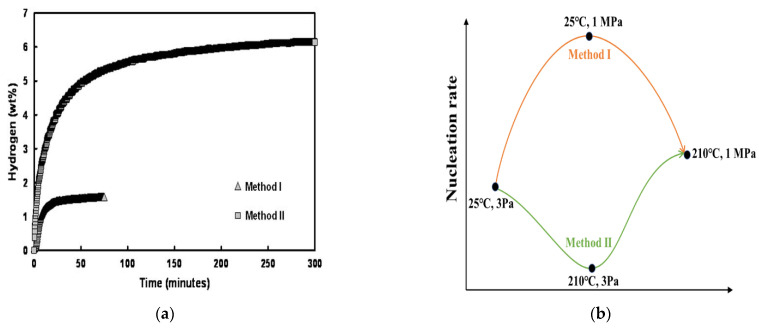

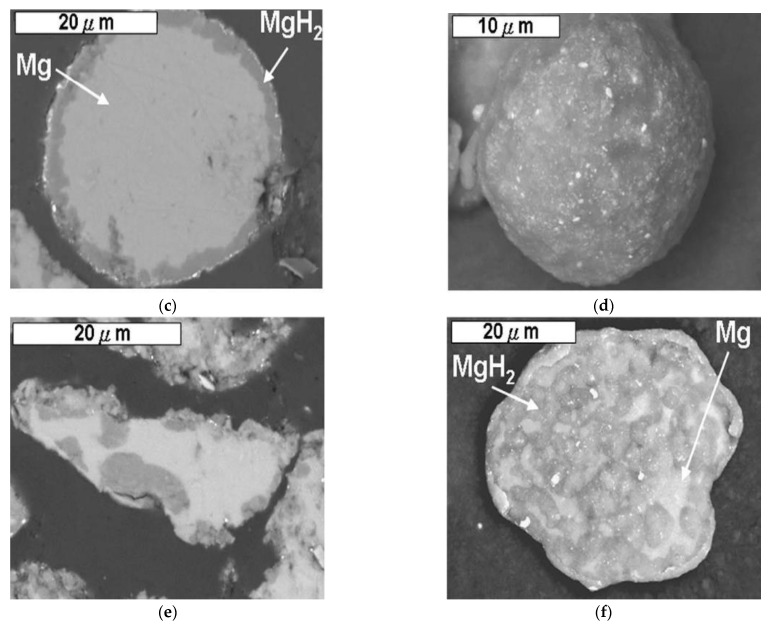
(**a**) Hydrogen absorption as a function of time for the two hydrogenation methods employed; (**b**) A scheme of the influence of temperature and hydrogen pressure on the nucleation rate of MgH_2_ during the hydrogenation of Mg. SEM pictures comparing the hydride formation behavior for powders hydrogenated by Method I for 75 min ((**c**,**d**) and by Method II for 3 min (**e**,**f**)). (**c**,**e**) are the cross-sectional views, and (**d**,**f**) are the external views of the powders [82].

As mentioned above, the nucleation and growth behavior of MgH_2_ during hydrogenation of Mg can exert a significant effect on both the hydrogen absorption kinetics and the hydrogen storage capacity, implying the importance of choosing an appropriate preparation method and kinetic test conditions for hydrogen absorption and desorption. Tien et al. [82] performed two hydrogenation methods on the Ni-coated pure Mg powder in order to investigate the effect of the hydride nucleation rate on the hydrogen storage properties of Mg. In Method I, the specimen chamber was first pressurized with hydrogen to 1 MPa, and then the temperature was raised at the approximate rate of 12 °C/min from room temperature to 210 °C. While the second approach, Method II, consisted of first heating the specimen chamber under the low partial pressure of hydrogen (approximately 3 Pa) up to 210 °C and then increasing the pressure to 1 MPa. It was found that the hydrogen capacity and hydrogen absorption speed are much higher for Method II than for Method I (Figure 11a). Considering the characteristics of the driving force for MgH_2_ nucleation and the observation that the saturation of the hydrogen absorption is achieved when approximately more than 80% of the powders have a surface coverage by hydride of 80% or more, Tien et al. [82] believed that the lower hydrogen capacity of the sample prepared by Method I arises from the low temperature in the early stage of Method I. This leads to a high nucleation rate, and hence the small MgH_2_ colonies/grains densely distributed on the surface (Figure 11c,d). Furthermore, the higher hydrogen capacity of the sample prepared by Method II arises from the high temperature in the early stage of Method II, which led to the low nucleation rate and hence the large MgH_2_ colonies/grains dispersed on the surface (Figure 11e,f). Thus, for a given particle geometry, there are optimum hydrogenation temperatures and pressure that lead to a near-theoretical hydrogen capacity with a fast absorption rate. A scheme of the influence of hydrogenation methods, performed in the work of Tien et al. [82], on the nucleation rate is presented in Figure 11b. It can be seen that the nucleation rate for Method I is higher than the nucleation rate for Method II during the hydrogenation process. 

The different nucleation behaviors of MgH_2_ can be explained considering that the thermodynamic parameters involved in the nucleation step, such as the difference in Gibbs free energy between the two phases and the interface energy, can be controlled by the reaction temperature and hydrogen gas pressure [63]. Especially, the Gibbs free energy differences between the two phases can explain the opposite behavior of MgH_2_ preferentially nucleating at low temperature and high pressure while Mg preferentially nucleating at high temperature and low pressure since the Gibbs free energy changes for these two processes are reversed. A long-range diffusion of metal atoms is required for the *β*-phase growth, which occurs as a result of the deposition of the *β*-phase from a supersaturated *α*-solid solution. The *β*-phase growth is known as diffusion-controlled in the case of fast transfer of atoms across the interface (the interfacial reaction), which makes the *β*-phase growth rate governed by the lattice-diffusion-induced removing rate of the excess atoms from the interface ahead. However, the *β*-phase growth is interface-controlled in the case of the much slower interfacial reaction than the lattice diffusion rate. The *β*-phase growth can also be mixed and controlled in the case of comparable rates of the interface reaction and the diffusion process [81]. In addition, the growth of the MgH_2_ phase was also reported to be controlled by the fast diffusion of hydrogen from the particle surface along the hydride–metal interface [83]. With the consideration of all of these factors, which control the growth of the MgH_2_ phase, the fact that the driving force of the MgH_2_ growth is the high temperature and pressure [10,71,82] can be explained. The facilitation of the high temperature for the long-range diffusion of metal atoms and the diffusion of hydrogen from the particle surface along the hydride–metal interface is of the same order for the hydrogenation (*β*-phase growth) and dehydrogenation (*α*-solid solution growth) reactions, respectively. Thus, the high temperature is also the driving force for Mg growth during MgH_2_ dehydrogenation.

**Figure 12:**
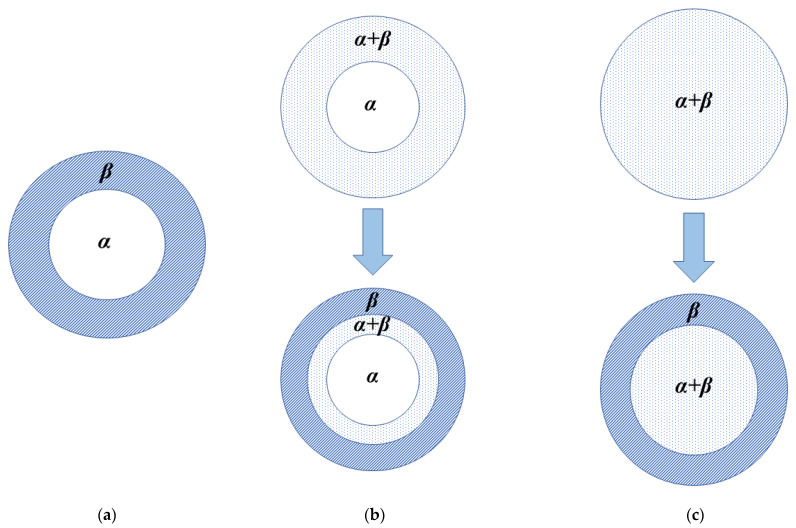
Model of hydriding kinetics. (**a**) model with no two-phase (*α* + *β*) region (continuous moving boundary); (**b**) model with a definite width of *α* + *β* two-phase region; (**c**) model of an entirely two-phase (*α* + *β*) region in its initial and later stages, respectively [81].

According to the readiness of hydride phase (*β*-phase) nucleation in the solid solution matrix (*α*-phase), three cases for the phase transformation process can be built [10,51,81] (Figure 12): (1) The two-phase coexisting region is absent (Figure 12a) with a quickly formed continuous *β*-phase layer on the outside of particles due to the easy and fast nucleation and growth of the *β*-phase in the *α*-matrix. This occurs when the volume expansion caused by phase transformation is low and nonequilibrium defects, such as excess vacancies, dislocations, grain boundaries, stacking faults, and inclusions, exist in the *α*-phase, leading to a low strain energy change. This also occurs when high hydrogen pressure and low temperature are applied because the low temperature allows a high nucleation rate. The high hydrogen pressure leads to a high hydrogen concentration gradient from the surface to the core, which causes preferential significant nucleation and growth on the surface. Then the *β*-phase grows toward the center of the supersaturation area, which is similar to the case described by the “shrinking unreacted core” model; (2) Three layers *α*, *α* + *β*, *β* coexist with the nucleation of the *β*-phase at multiple points inside the *α*-matrix (Figure 12b) when the *β*-phase nucleation is slow, but the *β*-phase growth is fast. This case occurs at a high temperature and high hydrogen pressure, with the high temperature predominating over the high hydrogen pressure in determining the nucleation rate. The high temperature allows a low nucleation rate, which leads to a wide region of hydrogen supersaturation in the matrix and, thereby, a simultaneous deposition of *β*-phase at multiple points in the supersaturation region, and the high hydrogen pressure-induced high hydrogen concentration gradient causes the preferential nucleation and growth on the surface (Figure 12b top). The continuous hydrogen diffusion from the surface to the center leads to the increasing width of the *β*-phase region and the decreasing width of the *α* + *β* two-phase region (Figure 12b bottom). It can be believed that if the growth of the *β*-phase is fast enough, the *α* + *β* phase region will disappear for a while, changing the pattern to the one in the first case (Figure 12a). However, the coexistence of three layers *α*, *α* + *β*, *β* should subsequently appear due to the slow nucleation of the *β*-phase; (3) The *β*-phase distributes throughout the matrix (Figure 12c) when the nucleation and growth of the *β*-phase are slow. This case occurs at a high temperature and low hydrogen pressure, with the low hydrogen pressure predominating over the high temperature in determining the growth rate. The low hydrogen pressure-induced low hydrogen concentration gradient and slow growth of the *β*-phase are unfavorable to the formation of the continuous *β*-phase layer on the outer surface. This leads to the fact that, unlike the situation presented in the second case (Figure 12b), the wide region of hydrogen supersaturation in the matrix caused by the high temperature expands all over the matrix. At a later stage, the hydrogen diffusion leads to the formation of the *β*-phase in the outer layer.

## 4. Conclusions

The variation of driving forces exert an influence not only on the hydrogenation/dehydrogenation kinetics but also on the corresponding reaction mechanisms. Experimentally observed nucleation and growth behavior of Mg/MgH_2_ during de/hydrogenation of MgH_2_/Mg is influenced by external factors approximately in the following ways: (1) During hydrogenation of Mg, low temperature and high hydrogen pressure allow fast nucleation of MgH_2_, while high temperature and hydrogen pressure facilitate the growth of MgH_2_ nuclei; (2) During dehydrogenation of MgH_2_, high temperature and low hydrogen pressure facilitate both fast nucleation and growth of Mg nuclei. Nucleation and growth behavior of Mg/MgH_2_ can also be influenced by the internal factors: (1) For pure MgH_2_ without pre-existing Mg grains, surface nucleation of Mg would be easier than nucleation of Mg within the volume, while there is a great deal of debate surrounding the situation in the presence of pre-existing Mg grains within the volume of MgH_2_; (2) Crystal defects present throughout the matrix lead to fast and easy nucleation and growth of the *β*-phase in the hydrogenation process. Different parameter values for both the external and internal factors lead to the complexity of the nucleation and growth behavior of Mg/MgH_2_, especially during the hydrogenation of Mg, which can be more varied after alloying or adding catalysts, thereby making the design of the Mg-based hydrogen storage materials using numerical simulation more difficult. In the future, a high-accuracy and concise nucleation and growth model, which has analytical formulas, is applicable under isothermal and non-isothermal conditions, and includes multiple factors, is desirable for further analysis of the nucleation and growth behavior during the hydrogenation and dehydrogenation of the hydrogen storage materials based on the Mg/MgH_2_ system. Moreover, the analysis of the nucleation and growth behavior may be improved by combining the kinetic analysis with in situ experiments or molecular dynamics theory.

## Data Availability

The raw/processed data required to reproduce these findings cannot be shared at this time as the data also forms part of an ongoing study.
